# Deciphering the evolutionary origin of the *RB1* imprint

**DOI:** 10.1186/1756-8935-6-S1-P38

**Published:** 2013-03-18

**Authors:** Deniz Kanber, Christian Roos, Jörg Gromoll, Bernhard Horsthemke, Dietmar Lohmann, Karin Buiting

**Affiliations:** 1 Institut für Humangenetik, Universitätsklinikum Essen, Essen, Germany; 2 Deutsches Primatenzentrum, Göttingen, Germany; 3 Centrum für Reproduktionsmedizin und Andrologie, Universitätsklinikum Münster, Münster, Germany

## 

Previously we could show that the *RB1* gene is imprinted. Skewed expression in favour of the maternal allele is due to a differentially methylated CpG-island within intron 2 of the *RB1* gene. This CpG-island (CpG85) serves as a promoter for an alternative *RB1* transcript and is part of a truncated processed pseudogene (*PPP1R26P1*), which is derived from the *PPP1R26* gene (previously *KIAA0649*) located on chromosome 9.

We could now narrow down the time interval of this retrotranspositional event by *in silico* analyses, which revealed that the ancestral gene *PPP1R26* is present in all primates, whereas the pseudogene copy within the *RB1* gene is only present in higher primates, which comprise Catarrhini (Old World Monkeys, Gibbons, Great Apes and Human) and Platyrrhini (New World Monkeys). Thus, the retrotransposition of *PPP1R26* has occurred after the divergence of Strepsirrhini and higher primates, but before the split between Catarrhini and Platyrrhini. Although information for Tarsiidae as distant sister lineage to higher primates is lacking, the retrotransposition of *PPP1R26* into the *RB1* gene appears to coincide with the retrotranspositional explosion described by Ohshima et al., 2003. Moreover, the *in silico* analysis revealed that there are additional pseudogene copies on chromosome 22 in human and chimp, which must have been derived from independent retrotransposition events. Only the chimp and the marmoset have another copy on chromosome 8 and chromosome 4, respectively.

For further examination of the evolutionary origin of the *RB1* imprint we compared the methylation patterns of the ancestral gene *PPP1R26* and its pseudogenes in different primates (human, chimp, rhesus, orangutan and marmoset). Methylation analysis by next generation bisulfite sequencing on the ROCHE/454 GS Junior showed that the pseudogene copy within the *RB1* gene is differentially methylated in all primates studied. All other copies are fully methylated except the additional copy on chromosome 4 in the marmoset, which seemed to be differentially methylated. By using an informative SNP for the methylation analysis in 8 individuals from 4 different families we could show that the methylation pattern of the copy on chromosome 4 in the marmoset is not parent-of-origin-specific, but allele-specific. We conclude that the epigenetic fate of a *PPP1R26* pseudogene after integration depends on the DNA sequence and selective forces at the integration site.

